# Are managers of emerging markets more opportunistic? application of Benford’s Law

**DOI:** 10.1371/journal.pone.0313611

**Published:** 2024-12-03

**Authors:** Shoaib Hassan, Muhammad Aksar, Maqbool Ahmad, Jana Kajanova

**Affiliations:** 1 Department of Management Sciences, National University of Modern Languages, Rawalpindi, Pakistan; 2 Faculty of Management, Department of Information Management and Business Systems, Comenius University Bratislava, Bratislava, Slovakia; 3 Faculty of Management, Department of Economics and Finance, Comenius University Bratislava, Bratislava, Slovakia; Universidad de Valladolid, SPAIN

## Abstract

**Purpose:**

The current research analyzes cosmetic earnings management practices in emerging and developed markets before and after the global financial crisis.

**Design/Methodology/Approach:**

Using digital analysis, by applying Benford’s Law the study analyzes the earnings adjustments that exceed a key reference point to determine whether earnings management anomaly exists or not? Based on a sample of 87165 firm-year observations of UK, US, Brazil, Russia, India, China and Pakistan listed corporations.

**Findings:**

Findings show that the managers of emerging markets have more incentive to manipulate earnings than their counterparts from developed markets. Further, the implementation of strict governance and legislative measures after the global financial crisis have significantly reduced the opportunistic behaviour of managers to manipulate earnings. However, the impact is lesser in emerging markets as compared to UK and US.

**Research implications:**

The empirical findings of this research are useful for policy making and regulatory authorities, investors and other stakeholders as our findings shed light on the restriction of cosmetic earnings management practices.

**Originality/Value:**

First study that tried to capture the cosmetic earnings management practices in both emerging and developed countries and in both pre and post financial crisis scenario.

## 1. Introduction

Concerns about the issues that emerge from information asymmetry make it difficult for investors to understand the firms’ actual performance [1]. This problem is more serious in emerging markets, thus creating more apprehension regarding the financial statements of such firms compared to those operating in developed countries [2–4]. Many studies suggest that accounting information is less accurate in emerging economies relative to developed countries [5]. High quality accounting information availability seems to play the most important role in mitigating information asymmetry and earnings management practices [6–8].

Earnings management refers to the practices by which managers of firms manipulate accounting figures to achieve targeted results [9–11]. According to Healy and Wahlen [12] managers use subjective judgment in recognizing transactions and in the financial reporting processes to manipulate financial reports. It is generally considered that earnings management results in fraudulent activities, and thus misleading stakeholders, even if the changes to the financial reports follow accounting principles and standards [13]. A reduction in the quality of financial statements is an obvious outcome of earnings management. Excessive earnings management can have serious implications in terms of corporate fraud. Thus, to reduce the chances for corporate fraud, many countries, including United States, United Kingdom, Brazil, Russia, India, China and Pakistan implemented regulations and laws to strengthen the governance and legislative system.

According to Burgstahler et al. [14], a country’s institutional arrangements greatly impact the extent of earnings management. Developed markets, compared to emerging markets, have established a more comprehensive legal system and investor protection policies. Man and Wong [15] observe lower earnings management in countries with stronger legal systems. Similarly, they find that countries that provide less protection to investors have excessive earnings management practices [16–18]. Hence, weak investor protection in developing markets is expected to provide more incentives to inside managers to manipulate the firm’s performance.

Past studies indicate two major categories of the critical determinants of Cosmetic Earnings Management (CEM). The first category holds zero as the threshold for earnings management. According to the empirical studies of Halaoua, Hamdi and Mejri [19], Park, Song and Lee [20] & Leuz et al. [21] firms avoid reported losses through earnings management and can thus cross over the zero threshold. The second category uses n × 10k as a key reference point to determine the threshold of CEM [22]. As for instance, if the firm’s expected net income is $10 million, but the actual earnings are only $9 million, then managers may adjust the data through digital rounding in such a way that they achieve the expected earnings. Benford’s law is a common method to investigate instances of digital rounding in financial data. Many studies use Benford’s law for a digital analysis, though there is still little research that analyzes and distinguishes between developed and emerging markets in this respect.

This study offers more evidence on the cosmetic earnings management of both the developed and emerging markets in many ways. Firstly, most of the previous research studies used accrual-based models to measure earnings management. The literature suggest that accruals models are criticized for estimation errors that may lead to conflicting results [23,24]. Moreover, the managers are mostly indulged in upward manipulation of earnings by rounding off the digits. Therefore, in this study, we used Benford’s law to analyze the cosmetic earnings management behaviour of managers.

Secondly, the prior research studies observed that the opportunistic behaviour of managers is different in emerging and developed markets. In case of institutions providing weak investor protection, there are more chances of low quality of earnings information and severe earnings management [25,26]. There are many studies which suggest that in the presence of strong legal protection for investors, the managers are less likely to engage in earnings manipulation [27,28]. The current study therefore compares developed and emerging economies to study the phenomenon of earnings management by employing Benford’s law. We analyze the earnings adjustments that exceed a key reference point to determine whether an earnings management anomaly exists or not.

Finally, we also observe whether the gradual strengthening of governance and legal framework by governments changed earnings management practices or not (by taking global financial crisis as cut-off point). Therefore, we aim to determine how earnings management differs between developed and emerging economies through a comparison of actual earnings numbers in the UK, US, India and Pakistan using Benford’s law.

Based on the findings of this research, it is observed that earnings management exists in both the emerging and developed markets. However, the managers of emerging markets have more incentive to manipulate earnings than their counterparts from developed markets. Moreover, the implementation of strict governance mechanism has significantly reduced the opportunistic behaviour of managers to manipulate earnings especially after the global financial crisis. The empirical findings of this research are useful for policy making and regulatory authorities, investors and other stakeholders as our findings shed light on the restriction of managerial behaviour caused by the improvement in governance and legislative mechanism. Like developed countries, the regulators of emerging economies are required to have extensive requirements of disclosure, legal protection of investors, creditors and shareholders to control managerial discretion.

The remaining paper proceed as follows: Section 2 presents the literature review and outlines research hypotheses. Section 3 outlines research methodology used in this research. Section 4 discuss the empirical findings and last section presents conclusion of study.

## 2. Literature review and hypothesis development

### 2.1. Benford’s Law and earnings management

It has been observed that earnings management is a matter of concern all around the world [29]. The managerial practices of earnings management include using such processes which can adjust or alter the data recorded in financial reports. The primary intention is to mislead stakeholders regarding corporate performance of the firm. Earnings management also explains the senior management’s contractual behavior regarding the usage of accounting and financial data [12]. This behavior of earnings management includes controlling transaction timings, selecting the timings of new accounting principles, adjusting discretionary accruals and selecting accounting standards.

All of the methods adopted by managers to engage in earnings management activities lead to affect the presentation of financial reports, thus misleading outside stakeholders since financial statements are the main source that help investors and creditors to develop a perception regarding the firm’s performance. Thus, adjusting figures in financial reports results in affecting the judgment of external stakeholders regarding the firm’s performance. The literature reports many studies which show that managers have incentives to manipulate earnings in order to reach the desired threshold [23]. An extensive increasing trend in the usage of accruals for detection or earnings management has been observed since the mid of the 1980s [30]. Jones and modified Jones model are the most popular ones among various accrual models [23]. Many researchers have used these models for detecting instances of earnings management. Whereas, there are also several studies which found that accrual models are not very strong in accurate detection of earnings management [23,24]. According to the definition given by Thomas [31] in his study, EM refers to small rounding off of reported earnings in an upward direction. This process generates more than actually reported zeros and less than expected nines as the second digit of income.

According to the findings of [3] Lin et al. (2011), managers engage in CEM because of two main reasons. As per the first reason, earning numbers relate to key cognitive reference points. The second reason is the use of the bonus, options contracts and lending, which encourages managers to round off the income figures once in a while. The present study attempts to detect CEM by analyzing the actual distribution of income figures. Recently, in academic literature as well as in practice, Benford’s Law has become a popular and most accepted tool for identifying contrived data [32]. There are a number of appealing characteristics for the examination of digits and their distribution in earning numbers for identification of earnings management.

Firstly, it is not required by researchers to determine the potentially noisy abnormal accruals. The other appealing characteristic is that a large number of potential earning manipulators can be identified by researchers without having to raise specific assumptions related to methods and motivation for earnings management [12]. As shown by Benford [33], the naturally occurring numbers have their expected distribution skewed towards *one* for the beginning digits (as *0* cannot be the first digit), similarly, it is *zero* for the second digit. The basis for numerically analyzing the sequential nature of numbers in a similar way is provided by Benford’s Law. The variations between actual and expected frequencies indicate that data has been manipulated [3]. Another study provided empirical evidence showing that corporate earnings are according to Benford’s Law when there is no earnings management [34]. Benford’s Law was further analyzed by Skousen et al. [35], who investigated data sets which followed the law and detected different types of accounting frauds.

Subsequently, in later studies, various analytical methods have been extended by researchers to analyze Benford’s Law. The studied methods included investigating the heaping anomaly [36] and increasing the number of digits used in the analysis [37]. Similarly, Diekmann, [38] studied the post-SOX environment and found out the degree to which financial statements are manipulated by firms through CEM. The aforementioned research showed that the financial statements reliability is enhanced and earnings management is constrained by the SOX respectfully. The summarized studies also show that for the analysis of EM behavior, Benford’s Law can be applied. Hence, this research employs Benford’s Law for investigating the level of variation in CEM. In this work, we use the UK and US to represent developed markets and Brazil, Russia, India, China and Pakistan as a representative of emerging markets.

### 2.2 Hypotheses development

According to Man and Wong [15], markets that provide robust legal protection for investors in their institutional environment are effective in controlling managers’ self-interest. Previous studies highlight that in the presence of stronger legal protection, managers are less likely to engage in earnings manipulation [39,40]. Another study concluded that real earnings management exist in Pakistani and UK based companies but big and small size firms of both economies real earnings management is significantly different [41]. Developed markets have extensive disclosure requirements, strong creditor and shareholders’ rights, and complete regulatory mechanisms; thus, managerial discretion is lower. We therefore hypothesize that in developed markets, the degree of CEM is lower than it is in emerging markets. We state the first hypothesis of this study formally as follows:

***H1*:** *Managers of non-financial firms in emerging markets manipulate earnings more than their counterparts in developed markets do*.

According to the discussion above, corporate governance mechanisms can successfully minimize earnings management behavior [25,26]. In addition, effective corporate governance also significantly reduces managers’ incentives to manipulate earnings. Since 2002, the emerging as well as developed economies implemented laws to strengthen corporate governance mechanisms. In their study, Cohen et al. [42] highlight that the corporate governance environment improved significantly in the Post-Sarbanes–Oxley era. The current study expects to find a decline in earnings management due to the ongoing enactment of corporate governance mechanisms since 2003. To investigate the differences before and after corporate governance mechanisms were strengthened, we divide the sample into two periods: before and after 2008. We state the second hypothesis of this study formally as follows:

***H2a*.** *The propensity for cosmetic earnings management in emerging and developed markets will be lower after the financial crisis*.

## 3. Methodology

### 3.1 Data

We analyzed the net income of non-financial firms listed of US, UK, Brazil, Russia, China, India and Pakistan. The secondary data is collected from the annual reports of respective non-financial firms for the years 1999 to 2018. After collecting the data, we removed values with negative net income, leaving a sample of 87165 (28254, 3731, 4086, 1706, 24944, 20504 and 3940) firm year observations for US, UK, Brazil, Russia, India, China and Pakistani listed firms respectively. In order to analyze the effectiveness of governance framework. We distributed the data into two groups (i.e. the pre and post-crisis periods) for both economies using 2008 as the cut-off point. Table 1 is showing the number of observations used for analysis purpose in each country.

**Table 1:** Observations used in the study.

	Full Sample	Pre-Crisis	Post Crisis	Between Pre and Post Crisis
UK	3731	1219	2417	95
USA	28254	14230	12126	1898
Pakistan	3940	2405	1460	75
India	24944	13119	11125	700
China	20504	11536	8650	318
Brazil	4086	1386	1576	1124
Russia	1706	216	1379	111
**Total**	**87165**	**44111**	**38733**	**4321**

### 3.2. Ethical declaration

We, the authors of this research paper, hereby declare that the study has been conducted in accordance with the highest ethical standards.

### 3.3. Research methodology

#### 3.3.1 Benford’s Law

The expected values for 10 digits from *zero* to *nine* from net income of both the developed and emerging markets sample countries listed firms are determined using Benford’s Law [43]. Benford [33] disclosed that for the 1^st^ digit, the expected proportion is started from 1 to 9, as the 1^st^ digit cannot be zero and the expected proportion can be a zero for the 2^nd^ digit. The methodology employed in the present study is adopted from previous study [44]. The expected proportion for digit b(b ∈{1,2,…,9} in Benford’s law is as follows:

$$Expected\;proportion\;(b\;digit)={log}_{10}(b+1)-{log}_{10}(b)$$ (1)

The above equation can be simplified as:

$$Expected\;proportion\;(b\;digit)={log}_{10}\left(1+\frac{1}{b}\right)$$ (2)

#### 3.3.2. Chi-square (χ^2^) test

The extension of the *z*-test is known as a χ^2^-test, which may be used for conformism of Benford’s Law [45]. The *z*-test is used to test one digit at a time, but the χ^2^-test combines the results for testing of each expected digit with each actual digit in one test statistic. The χ^2^-test is determined as:

For the 1^st^ digit Eq 3:

$$Chi-square=\sum_{i=1}^{9}\frac{{\left[{nP}_{o}-{nP}_{e}\right]}^{2}}{{nP}_{e}}$$ (3)

For the other digits Eq 4:

$$Chi-square=\sum_{i=0}^{9}\frac{{\left[{nP}_{0}-{nP}_{e}\right]}^{2}}{{nP}_{e}}$$ (4)

Where *P*_*e*_ and *P*_*o*_ are the expected and observed (actual) proportions, respectively, and *n* is the sample size.

#### 3.3.3. *Z* Statistic

To test the significance of the deviation of the expected proportion from the actual proportion in numbers from 1 to 9 for the 1^st^ digit and from 1 to 9 for other digits are tested by using the *z*-test. The null hypothesis, that the expected proportion does not significantly differ from the actual proportion, is rejected if the *z* calculated value falls in the rejected area at the 10%, 5% and 1% significance levels with the tabulated values of 1.64, 1.96 and 2.57 respectively.


$$Z_{cal}=\frac{|P_{0}-P_{e}|-\frac{1}{2n}}{\sqrt{\frac{P_{o}(1-P_{0})}{n}}}$$ (5)

#### 3.3.4. Cramer’s V-test

Cramer’s V-test (CV-test) is taken into account to find out the degree of the association after the significance of the χ^2^. The χ^2^-test just elaborates the significance of the association, but the CV-test elaborates that up to what extent the association exists. The CV-test has values from 0 to 1, and the value closer to zero expresses a weak relationship between the variables and a strong relationship if the value is closer to 1. The following equation is used for the CV-test.


$$Cramer's\;V=\sqrt{\frac{Chi-square}{n(k-1)}}$$ (6)

Where *n* is the sample size and *k* is the number of variables.

## 4. Empirical results

Tables 2–8 present the results on the application of Benford’s Law. More precisely, these tables report the results of CEM for the non-financial firms operating in the UK, US, Brazil, Russia, India, China and Pakistan, respectively. The distribution of each number from 1 to 9 for the 1^st^ leading digit and from 0 to 9 for the 2^nd^ and the 3^rd^ leading digits of positive net income of the non-financial firms of aforementioned countries is explained in detail. The first row of each table represents the observed proportion (percentage of the sample) of each number (1 to 9) of leading digits (1 to 3). In the second row, the expected proportion determined by Benford’s Law has been mentioned. The 3^rd^ row depicts the percentage deviation rate for each number (1 to 9) of leading digits (1 to 3). The rate is determined by taking the difference between the actual and the expected proportion and dividing the results by the expected proportion. The final row illustrates results of the *z* statistic. In each table, the results for χ^2^ of leading digits (1 to 3) are determined to check whether managers have more incentives by manipulating the earnings in developed economies (UK and US) or emerging markets (such as Brazil, Russia, India, China and Pakistan). According to Halaoua, Hamdi, & Mejri [19] results for the χ^2^ are affected by sample size. Therefore, to control the effect of sample size, another test, i.e., CV-test, is used to compare the variations in different level of Benford’s Law.

**Table 2:** From 1^st^ to 3^rd^ digits Benford’s Law on net annual income (UK sample).

	Number	0	1	2	3	4	5	6	7	8	9	χ^2^	Cramer’s V
**1**^**st**^ **digit**	**Observed %**		30.72	16.4	12.25	10.53	7.34	6.94	5.79	5.33	4.69	8.88	0.017
***N* = 3731**	**Expected %**		30.1	17.61	12.49	9.69	7.92	6.7	5.8	5.12	4.58		
	**Deviation rate**		2.05	-6.85	-1.93	8.7	-7.27	3.61	-0.18	4.17	2.41		
	***Z* statistic**		0.81	**1.99** **	0.45	**1.68** *	1.35	0.58	0.03	0.58	0.32		
**2**^**nd**^ **digit**	**Observed %**	12.98	10.99	10.57	10.54	9.92	9.52	9.36	8.53	9.31	8.34	6.32	0.014
***N* = 3731**	**Expected %**	11.97	11.39	10.88	10.43	10.03	9.67	9.34	9.04	8.76	8.5		
	**Deviation rate**	8.43	-3.47	-2.89	1.05	-1.07	-1.55	0.2	-5.67	6.23	-1.88		
	***Z* statistic**	**1.83** *	0.77	0.62	0.22	0.22	0.31	0.04	1.12	1.15	0.35		
**3**^**rd**^ **digit**	**Observed %**	13.05	10.08	9.7	10.24	9.81	9.51	9.73	9.57	9.65	8.66	**26.11** ***	0.028
***N* = 3731**	**Expected %**	10.18	10.14	10.1	10.06	10.02	9.98	9.94	9.9	9.86	9.83		
	**Deviation rate**	28.22	-0.61	-3.94	1.77	-2.1	-4.66	-2.12	-3.35	-2.14	-11.93		
** **	***Z* statistic**	**5.21** ***	0.13	0.82	0.36	0.43	0.97	0.43	0.69	0.44	**2.55** ***		

Note: Observed and expected values have been determined in the percentage of the sample (*N*). Deviation rate has been calculated by taking the difference of observed and expected percentage values divided by the expected percentage value.

*, ** and *** indicate significance at 10%, 5% and 1% levels, respectively.

**Table 3:** From 1st to 3rd digits Benford’s Law on net annual income companies in USA.

	Number	0	1	2	3	4	5	6	7	8	9	Chi-Square	Cramer’s V
**1st digit**	**Observed Percentage**		29.18	18.04	13.47	11.59	6.98	6.59	6.37	5.07	5.16	170.00	0.027
**n = 28254**	**Expected Percentage**		30.1	17.61	12.49	9.69	7.92	6.7	5.8	5.12	4.58		
	**Deviation Rate**		-3.06	2.46	7.88	19.57	-11.91	-1.57	9.80	-1.04	12.65		
	**Z-Stat**		1.24	0.69	1.76	**3.62** ***	**2.26** **	0.26	1.42	0.15	1.60		
**2nd digit**	**Observed Percentage**	13.76	11.54	10.99	12.12	10.22	9.81	9.83	8.78	9.77	8.59	196.30	0.028
**n = 28254**	**Expected Percentage**	11.97	11.39	10.88	10.43	10.03	9.67	9.34	9.04	8.76	8.5		
	**Deviation Rate**	14.94	1.36	1.00	16.20	1.89	1.40	5.21	-2.84	11.54	1.06		
	**Z-Stat**	**3.17** ***	0.30	0.21	3.16	0.38	0.28	1.00	0.55	2.08	0.20		
**3rd digit**	**Observed Percentage**	13.57	11.59	10.09	11.77	10.20	9.80	10.12	9.86	10.13	9.00	451.40	0.042
**n = 28254**	**Expected Percentage**	10.18	10.14	10.1	10.06	10.02	9.98	9.94	9.9	9.86	9.83		
	**Deviation Rate**	33.35	14.29	-0.09	17.04	1.82	-1.80	1.80	-0.45	2.75	-8.41		
	**Z-Stat**	**6.05** ***	2.77	0.02	**3.25** ***	0.37	0.37	0.36	0.09	0.55	**1.76** *		

Note: Observed and expected values have been determined in percentage of sample (n). Deviation rate has been calculated by taking difference of observed and expected percentage values divided by expected percentage value.

*, **, ***are at significant level 10%, 5% and 1% respectively.

**Table 4:** From 1^st^ to 3^rd^ digits Benford’s Law on net annual income (Pakistani sample).

	Number	0	1	2	3	4	5	6	7	8	9	χ2	Cramer’s V
**1**^**st**^ **digit**	**Observed Percentage**		31.03	17.6	12.75	9.96	7.88	6.14	5.48	4.67	4.48	6.32	0.014
***N* = 3940**	**Expected Percentage**		30.1	17.61	12.49	9.69	7.92	6.7	5.8	5.12	4.58		
	**Deviation Rate**		3.1	-0.08	2.09	2.8	-0.5	-8.31	-5.48	-8.7	-2.21		
	**Z-Stat**		1.23	0.02	0.48	0.55	0.09	1.42	0.85	1.29	0.3		
**2**^**nd**^ **digit**	**Observed Percentage**	13.24	10.87	10.35	10.79	9.74	9.37	8.81	8.98	8.93	8.91	10.56	0.017
***N* = 3940**	**Expected Percentage**	11.97	11.39	10.88	10.43	10.03	9.67	9.34	9.04	8.76	8.5		
	**Deviation Rate**	10.61	-4.6	-4.85	3.48	-2.89	-3.07	-5.67	-0.64	1.97	4.81		
	**Z-Stat**	**2.29** **	1.03	1.06	0.71	0.6	0.62	1.14	0.12	0.37	0.88		
**3**^**rd**^ **digit**	**Observed Percentage**	18.55	9.96	9.5	9.25	9.01	8.61	9.08	8.37	8.79	8.88	**196.68** ***	0.074
***N* = 3940**	**Expected Percentage**	10.18	10.14	10.1	10.06	10.02	9.98	9.94	9.9	9.86	9.83		
	**Deviation Rate**	82.23	-1.77	-5.98	-8.04	-10.12	-13.68	-8.65	-15.45	-10.89	-9.62		
** **	**Z-Stat**	**13.15** ***	0.37	1.26	**1.70** *	**2.16** **	**2.97** **	**1.83** *	**3.37** ***	**2.32** **	**2.03** **		

Note: Observed and expected values have been determined in the percentage of the sample (*N*). Deviation rate has been calculated by taking the difference of observed and expected percentage values divided by the expected percentage value.

*, ** and *** indicate significance at 10, 5 and 1% levels, respectively.

**Table 5:** From 1st to 3rd digits Benford’s Law on net annual income companies in Brazil.

	Number	0	1	2	3	4	5	6	7	8	9	Chi-Square	Cramer’s V
**1st digit**	**Observed Percentage**		40.55	19.33	12.16	7.98	5.14	4.50	4.04	2.67	3.62	374.98	0.107
**n = 4086**	**Expected Percentage**		30.1	17.61	12.49	9.69	7.92	6.7	5.8	5.12	4.58		
	**Deviation Rate**		34.73	9.79	-2.61	-17.66	-35.11	-32.79	-30.38	-47.90	-20.91		
	**Z-Stat**		**13.00** ***	**2.67** ***	0.61	**3.86** ***	**7.69** ***	**6.47** ***	**5.47** ***	**9.30** ***	**3.13** ***		
**2nd digit**	**Observed Percentage**	20.44	8.64	8.54	8.93	9.18	8.30	8.42	8.81	9.50	8.81	235.15	0.080
**n = 4086**	**Expected Percentage**	11.97	11.39	10.88	10.43	10.03	9.67	9.34	9.04	8.76	8.5		
	**Deviation Rate**	70.72	-24.15	-21.49	-14.35	-8.50	-14.20	-9.86	-2.54	8.40	3.65		
	**Z-Stat**	**12.82** ***	**5.98** ***	**5.11** ***	**3.21** ***	**1.80** *	**3.04** ***	**2.03** **	0.49	1.53	0.67		
**3rd digit**	**Observed Percentage**	15.64	9.81	9.64	9.57	8.98	9.35	9.30	9.52	8.54	9.64	97.73	0.051
**n = 4086**	**Expected Percentage**	10.18	10.14	10.1	10.06	10.02	9.98	9.94	9.9	9.86	9.83		
	**Deviation Rate**	53.62	-3.21	-4.53	-4.88	-10.36	-6.32	-6.44	-3.84	-13.37	-1.91		
** **	**Z-Stat**	**9.18** ***	0.67	0.95	1.02	**2.22** **	1.32	1.35	0.79	**2.88** ***	0.39		

Note: Observed and expected values have been determined in the percentage of the sample (*N*). Deviation rate has been calculated by taking the difference of observed and expected percentage values divided by the expected percentage value.

*, ** and *** indicate significance at 10, 5 and 1% levels, respectively.

**Table 6:** From 1st to 3rd digits Benford’s Law on net annual income companies in Russia.

	Number	0	1	2	3	4	5	6	7	8	9	Chi-Square	Cramer’s V
**1st digit**	**Observed Percentage**		13.44	7.61	5.43	3.16	3.33	2.47	2.47	2.03	1.69	1406.00	0.321
**n = 1706**	**Expected Percentage**		30.1	17.61	12.49	9.69	7.92	6.7	5.8	5.12	4.58		
	**Deviation Rate**		-55.36	-56.78	-56.50	-67.42	-57.97	-63.11	-57.38	-60.33	-63.13		
	**Z-Stat**		**29.85** ***	**23.03** ***	**19.02** ***	**22.82** ***	**15.63** ***	**16.63** ***	**13.09** ***	**13.37** ***	**13.71** ***		
**2nd digit**	**Observed Percentage**	4.31	4.31	4.31	4.31	4.31	4.31	4.31	4.31	4.31	4.31	1331.00	0.294
**n = 1706**	**Expected Percentage**	11.97	11.39	10.88	10.43	10.03	9.67	9.34	9.04	8.76	8.5		
	**Deviation Rate**	-64.02	-62.18	-60.41	-58.70	-57.05	-55.46	-53.88	-52.35	-50.83	-49.32		
	**Z-Stat**	**23.05** ***	**21.31** ***	**19.77** ***	**18.42** ***	**17.22** ***	**16.13** ***	**15.14** ***	**14.24** ***	**13.40** ***	**12.61** ***		
**3rd digit**	**Observed Percentage**	1.71	4.53	4.50	4.06	4.36	4.33	4.36	3.99	5.16	4.63	1797.00	0.342
**n = 1706**	**Expected Percentage**	10.18	10.14	10.1	10.06	10.02	9.98	9.94	9.9	9.86	9.83		
	**Deviation Rate**	-83.17	-55.35	-55.41	-59.62	-56.52	-56.59	-56.17	-59.70	-47.63	-52.94		
** **	**Z-Stat**	**39.85** ***	**16.49** ***	**16.49** ***	**18.56** ***	**16.95** ***	**16.95** ***	**16.71** ***	**18.45** ***	**12.96** ***	**15.13** ***		

Note: Observed and expected values have been determined in the percentage of the sample (*N*). Deviation rate has been calculated by taking the difference of observed and expected percentage values divided by the expected percentage value.

*, ** and *** indicate significance at 10, 5 and 1% levels, respectively.

**Table 7:** From 1st to 3rd digits Benford’s Law on net annual income companies in China.

	Number	0	1	2	3	4	5	6	7	8	9	Chi-Square	Cramer’s V
**1st digit**	**Observed Percentage**		44.93	19.33	11.24	7.58	5.58	4.20	2.82	2.50	1.82	312.00	0.044
**n = 20504**	**Expected Percentage**		30.1	17.61	12.49	9.69	7.92	6.7	5.8	5.12	4.58		
	**Deviation Rate**		49.28	9.76	-10.03	-21.79	-29.55	-37.33	-51.31	-51.13	-60.28		
	**Z-Stat**		**18.21** ***	**2.66** ***	**2.42** ***	**4.87** ***	**6.23** ***	**7.62** ***	**10.97** ***	**10.24** ***	**12.62** ***		
**2nd digit**	**Observed Percentage**	10.34	9.78	10.20	9.65	9.98	10.10	9.70	9.82	9.66	9.87	16.00	0.009
**n = 20504**	**Expected Percentage**	11.97	11.39	10.88	10.43	10.03	9.67	9.34	9.04	8.76	8.5		
	**Deviation Rate**	-13.62	-14.10	-6.22	-7.46	-0.51	4.40	3.86	8.66	10.24	16.13		
	**Z-Stat**	**3.27** ***	**3.30** ***	1.37	**1.61** *	0.10	0.86	0.74	**1.61** *	**1.85** *	**2.81** ***		
**3rd digit**	**Observed Percentage**	0.88	55.65	55.80	55.51	56.14	57.64	53.28	55.82	55.97	54.94	89.00	0.022
**n = 20504**	**Expected Percentage**	10.18	10.14	10.1	10.06	10.02	9.98	9.94	9.9	9.86	9.83		
	**Deviation Rate**	-91.35	448.85	452.48	451.76	460.31	477.51	436.01	463.89	467.66	458.94		
** **	**Z-Stat**	**142.46** ***	**131.16** ***	**131.75** ***	**130.93** ***	**133.07** **	**138.08** ***	**124.36** ***	**132.40** **	**132.99** ***	**129.81** ***		

Note: Observed and expected values have been determined in the percentage of the sample (*N*). Deviation rate has been calculated by taking the difference of observed and expected percentage values divided by the expected percentage value.

*, ** and *** indicate significance at 10, 5 and 1% levels, respectively.

**Table 8:** From 1st to 3rd digits Benford’s Law on net annual income of Indian companies.

	Number	0	1	2	3	4	5	6	7	8	9	Chi-Square	Cramer’s V
**1st digit**	**Observed Percentage**		42.49	19.56	11.05	8.02	5.67	4.37	3.56	3.00	2.28	1300.00	0.081
**n = 24944**	**Expected Percentage**		30.1	17.61	12.49	9.69	7.92	6.7	5.8	5.12	4.58		
	**Deviation Rate**		41.15	11.05	-11.54	-17.26	-28.37	-34.78	-38.55	-41.35	-50.19		
	**Z-Stat**		**15.31** ***	**3.00** **	**2.81** ***	**3.76** ***	**5.93** ***	**6.96** ***	**7.37** ***	**7.58** ***	**9.40** ***		
**2nd digit**	**Observed Percentage**	10.57	9.86	10.07	10.22	10.02	10.00	10.11	9.79	9.70	9.68	79.00	0.019
**n = 24944**	**Expected Percentage**	11.97	11.39	10.88	10.43	10.03	9.67	9.34	9.04	8.76	8.5		
	**Deviation Rate**	-11.71	-13.47	-7.49	-2.02	-0.06	3.37	8.22	8.26	10.71	13.87		
	**Z-Stat**	**2.78** ***	**3.14** ***	1.65	0.42	0.01	0.66	1.55	1.53	**1.94** *	**2.44** ***		
**3rd digit**	**Observed Percentage**	2.94	11.61	11.97	11.76	11.77	11.84	11.77	11.61	11.33	3.40	2202.00	0.099
**n = 24944**	**Expected Percentage**	10.18	10.14	10.1	10.06	10.02	9.98	9.94	9.9	9.86	9.83		
	**Deviation Rate**	-71.09	14.49	18.52	16.87	17.50	18.63	18.36	17.26	14.90	-65.37		
** **	**Z-Stat**	**26.15** ***	**2.80** ***	**3.52** ***	**3.22** ***	**3.32** ***	**3.51** ***	**3.46** ***	**3.26** ***	**2.83** ***	**21.65** ***		

Note: Observed and expected values have been determined in the percentage of the sample (*N*). Deviation rate has been calculated by taking the difference of observed and expected percentage values divided by the expected percentage value.

*, ** and *** indicate significance at 10, 5 and 1% levels, respectively.

As it is evident in Tables 2 & 3, the results of *z* statistic for the US, and UK based companies are statistically significant at the number *zero* in the 2^nd^ and 3^rd^ digits, and the deviation rate is also positive, so the number *zero* in leading digits 2 and 3 is more frequented than the expected frequency. Numbers 2 and 4 at the 1^st^ leading digit are also more frequented than the expected frequency. Number 9 at the 3^rd^ leading digit is also more frequented than the expected frequency. The value of the χ^2^-test is only statistically significant at the 3^rd^ digit, suggesting that in the UK managers have more incentives by manipulating the earnings at the 3^rd^ digit. Likewise, the value of the χ^2^-test is only statistically significant at the 2^nd^ digit, suggesting that in the US managers use CEM by manipulating the earnings at the 2^nd^ digit.

Moving next to Table 4, the results of *z* statistic for the Pakistani listed firms are statistically significant at the number *zero* for the 2^nd^ and 3^rd^ digits and the deviation rate is positive, which constitute that the number *zero* has more observed frequencies than the expected frequencies. In the Pakistani context, it is also observed that at the 3^rd^ digit, the numbers from 2 to 9 are fewer frequented than the expected frequencies, as the deviations at these stages are negative and the *z* statistics are statistically significant. The value of the χ^2^-test at digit 3 is statistically significant, which means that Pakistani managers have more incentives by manipulating the earnings at the 3^rd^ digit. In both of the economies, managers have more incentives by manipulating the 3^rd^ digit of earnings.

According to Table 5, the results of *z* statistic for the Brazilian listed firms are statistically significant at the number *zero* for the 2^nd^ and 3^rd^ digits and the deviation rate is positive, which constitute that the number *zero* has more observed frequencies than the expected frequencies.

In the Brazilian context, it is also observed that at the 1^st^ digit, the numbers from 1 to 9 are more frequented than the expected frequencies, as the deviations at these stages are positive and the *z* statistics are statistically significant. At 2^nd^ digit, the numbers are more frequented from 0 to 5. Likewise, the numbers are more frequented at 4 and 8 in the 3^rd^ digit analysis. The value of the χ^2^-test at digit 1 and 2 is statistically significant, which means that Brazilian managers have more incentives by manipulating the earnings at the 1^st^ and 2^nd^ digit.

Table 6 represents the results of *z* statistic for the Russian listed firms which are statistically significant at the number *zero* for the 2^nd^ and 3^rd^ digits and the deviation rate is positive, which constitute that the number *zero* has more observed frequencies than the expected frequencies. In the Russian context, it is also observed that at the 1^st^, 2^nd^ and 3^rd^ digits, the numbers from 1 to 9 are more frequented than the expected frequencies, as the deviations at these stages are positive and the *z* statistics are statistically significant. The value of the χ^2^-test at 1^st^, 2^nd^ and 3^rd^ digits is statistically significant, which means that managers in these firms have more incentives by manipulating the earnings at all the levels.

As per the results of Table 7, the results of *z* statistic for the Chinese listed firms are statistically significant at the number *zero* for the 2^nd^ and 3^rd^ digits and the deviation rate is positive, which constitute that the number *zero* has more observed frequencies than the expected frequencies. In the Chinese firms, it is also observed that at the 1^st^ and 3^rd^ digits, the numbers from 1 to 9 are more frequented than the expected frequencies, as the deviations at these stages are positive and the *z* statistics are statistically significant. The value of the χ^2^-test at 1^st^ and 3^rd^ digits is statistically significant, which means that managers in these firms have more incentives by manipulating the 1^st^ and 3^rd^ digits of earnings.

Table 8 shows the results of *z* statistic for the Indian listed firms are statistically significant at the number *zero* for the 1^st^ and 3^rd^ digits and the deviation rate is positive, which constitute that the number *zero* has more observed frequencies than the expected frequencies. In the Chinese firms, it is also observed that at the 1^st^ and 3^rd^ digits, the numbers from 1 to 9 are more frequented than the expected frequencies, as the deviations at these stages are positive and the *z* statistics are statistically significant. The value of the χ^2^-test at 1^st^ and 3^rd^ digits is statistically significant, which means that managers in these firms have more incentives by manipulating the 1^st^ and 3^rd^ digits of earnings. As mentioned above, the CV-test is applied to control the effect of sample size which suggests that Cramer’s V values at the 2^nd^ and 3^rd^ digits determined in Pakistani and Russian context are greater than the values determined in the UK. Overall, our results constitute that in emerging economies (BRIC and Pakistan) the managers have higher incentives for manipulating the earnings by exaggerating the earnings digits.

### 4.1 Pre and post global financial crisis period analysis

Tables 9 and 10 represents the results for the pre and post-crisis period in the context of the developed countries. Table 9 represents that the *z* statistic for UK is significant at the numbers 1, 2 and 7 in the 1^st^ digit during the pre-crisis period. However, in the 1^st^ digit, no significant change is observed during the post-crisis period. In the 2^nd^ digit, the pre-crisis period is showing a significant change in the number *zero*. In the 2^nd^ digit for the post-crisis period the *z* statistic is significant for the number 8. The positive deviation reveals that observed values are more frequent than expected values and negative deviation rate shows that observed frequencies are fewer than expected frequencies. In the 3^rd^ digit, during both the pre- and post-crisis period, the *z* statistic is significant for the numbers *zero* and 9. The value of the χ^2^-test illustrates that the 3^rd^ digit is statistically significant for the post-crisis period. Thus, managers of the UK based firms have more incentives to manipulate the earnings at the 3^rd^ digit for the post-crisis period. The results for the Cramer’s V suggest that for all digits (1 to 3), it has more values for the pre-crisis period, so more variation is seen during the pre-crisis period in the context of the UK.

**Table 9:** Pre and post crisis (UK based companies) from 1^st^ to 3^rd^ digits Benford’s Law on net annual income of companies in UK.

	Number	0	1	2	3	4	5	6	7	8	9	χ^2^	Cramer’s V
**1**^**st**^ **digit; *N* = 1219**	**Observed %**		32.40	15.83	13.37	10.83	6.89	6.48	4.68	5.5	4.02	13.12	0.037
**Pre-crisis**	**Expected %**		30.10	17.61	12.49	9.69	7.92	6.7	5.8	5.12	4.58		
	**Deviation rate**		7.65	-10.09	7.06	11.75	-12.99	-3.27	-19.38	7.35	-12.23		
	***Z* statistic**		**1.72** *	**1.70** *	0.90	1.28	1.42	0.31	**1.86** *	0.58	1.00		
**1**^**st**^ **digit; *N* = 2417**	**Observed %**		29.90	16.68	11.7	10.39	7.56	7.17	6.33	5.25	5.02	7.18	0.018
**Post-crisis**	**Expected %**		30.10	17.61	12.49	9.69	7.92	6.7	5.8	5.12	4.58		
	**Deviation rate**		-0.68	-5.28	-6.29	7.23	-4.5	6.95	9.13	2.63	9.52		
** **	***Z* statistic**		0.22	1.25	1.23	1.15	0.67	0.90	1.09	0.30	1.00		
**2**^**nd**^ **digit; N = 1219**	**Observed %**	14.36	11.16	11.81	9.93	9.43	9.43	9.35	8.2	8.12	8.2	8.29	0.027
**Pre-crisis**	**Expected %**	11.97	11.39	10.88	10.43	10.03	9.67	9.34	9.04	8.76	8.5		
	**Deviation rate**	19.93	-2.05	8.58	-4.83	-5.94	-2.44	0.13	-9.25	-7.29	-3.49		
	***Z* statistic**	**2.38** ***	0.26	1.01	0.59	0.71	0.28	0.01	1.06	0.82	0.38		
**2**^**nd**^ **digit; N = 2417**	**Observed %**	12.3	10.91	9.95	10.83	10.15	9.55	9.36	8.68	9.87	8.4	6.9	0.018
**Post-crisis**	**Expected %**	11.97	11.39	10.88	10.43	10.03	9.67	9.34	9.04	8.76	8.5		
	**Deviation rate**	2.76	-4.23	-8.53	3.82	1.21	-1.2	0.16	-4	12.7	-1.18		
** **	***Z* statistic**	0.5	0.78	1.55	0.64	0.2	0.2	0.03	0.64	**1.87** *	0.18		
**3**^**rd**^ **digit; *N* = 1219**	**Observed %**	13.29	9.68	10.09	9.84	9.84	9.6	10.01	9.68	9.6	8.37	9.57	0.029
**Pre-crisis**	**Expected %**	10.18	10.14	10.1	10.06	10.02	9.98	9.94	9.90	9.86	9.83		
	**Deviation rate**	30.55	-4.54	-0.10	-2.15	-1.76	-3.83	0.69	-2.22	-2.66	-14.88		
	***Z* statistic**	**3.20** ***	0.54	0.01	0.25	0.21	0.45	0.08	0.26	0.31	**1.84** *		
**3**^**rd**^ **digit; *N* = 2417**	**Observed %**	12.94	10.27	9.51	10.43	9.79	9.47	9.59	9.51	9.67	8.80	17.65	0.028
**Post-crisis**	**Expected %**	10.18	10.14	10.10	10.06	10.02	9.98	9.94	9.90	9.86	9.83		
	**Deviation rate**	27.09	1.29	-5.80	3.68	-2.27	-5.06	-3.48	-3.90	-1.89	-10.5		
** **	***Z* statistic**	**4.12** ***	0.22	1.00	0.61	0.38	0.86	0.59	0.66	0.32	**1.83** *		

Note: Observed and expected values have been determined in the percentage of the sample (*N*). Deviation rate has been calculated by taking the difference of observed and expected percentage values divided by the expected percentage value.

*, ** and *** indicate significance at 10, 5 and 1% levels, respectively.

**Table 10:** Pre and post crisis (USA based companies) from 1st to 3rd digits Benford’s Law on net annual income of companies in USA.

	Number	0	1	2	3	4	5	6	7	8	9	Chi-Square	Cramer’s V
**1st digit n = 14230**	**Observed Percentage**		39.49	17.46	9.78	7.13	4.88	3.73	3.15	2.41	1.98	1674.40	0.121
**Pre-Crisis**	**Expected Percentage**		27.09	15.85	11.24	8.72	7.13	6.03	5.22	4.61	4.12		
	**Deviation Rate**		41.19	9.14	-11.73	-16.37	-28.41	-34.40	-35.71	-42.84	-46.86		
	**Z-Stat**		**8.72** ***	1.42	**1.64** *	**2.05** **	**3.47** ***	**4.04** ***	**3.93** ***	**4.74** ***	**5.11** ***		
**1st digit n = 12126**	**Observed Percentage**		36.85	17.76	10.13	7.31	5.36	4.16	3.27	3.02	2.14	938.70	0.098
**Post Crisis**	**Expected Percentage**		27.09	15.85	11.24	8.72	7.13	6.03	5.22	4.61	4.12		
	**Deviation Rate**		32.43	10.83	-8.89	-14.60	-22.35	-27.86	-33.57	-30.99	-43.30		
** **	**Z-Stat**		**9.95** ***	**2.40** ***	**1.76** *	**2.60** ***	**3.75** ***	**4.45** ***	**5.21** ***	**4.41** ***	**6.53** ***		
**2nd digit n = 14230**	**Observed Percentage**	9.82	9.00	8.99	8.94	9.02	9.14	9.14	8.74	8.66	8.54	72.34	0.023
**Pre-Crisis**	**Expected Percentage**	10.77	10.25	9.79	9.39	9.03	8.70	8.41	8.14	7.88	7.65		
	**Deviation Rate**	-7.99	-10.98	-7.35	-4.24	-0.10	4.49	7.82	6.73	8.91	10.53		
	**Z-Stat**	1.07	1.45	0.93	0.52	0.01	0.50	0.84	0.72	0.92	1.07		
**2nd digit n = 12126**	**Observed Percentage**	9.18	8.73	9.13	9.47	9.03	8.84	9.05	8.88	8.80	8.89	88.74	0.028
**Post Crisis**	**Expected Percentage**	10.77	10.25	9.79	9.39	9.03	8.70	8.41	8.14	7.88	7.65		
	**Deviation Rate**	-13.34	-13.38	-6.07	0.84	-0.01	1.44	6.92	8.20	10.43	14.64		
** **	**Z-Stat**	**2.65** ***	**2.58** ***	1.10	0.14	0.00	0.23	1.08	1.25	1.54	**2.09** **		
**3rd digit n = 14230**	**Observed Percentage**	2.65	10.45	10.77	10.58	10.60	10.66	10.59	10.45	10.20	3.06	2336.40	0.135
**Pre-Crisis**	**Expected Percentage**	9.16	9.13	9.09	9.05	9.02	8.98	8.95	8.91	8.87	8.85		
	**Deviation Rate**	-63.98	13.04	16.67	15.18	15.75	16.77	16.53	15.54	13.41	-58.84		
	**Z-Stat**	**13.45** ***	1.44	**1.81** **	**1.66** *	**1.71** *	**1.81** *	**1.78** *	**1.68** *	1.46	**11.14** ***		
**3rd digit n = 12126**	**Observed Percentage**	2.90	9.64	9.96	10.23	10.11	9.54	9.45	9.57	9.38	9.20	1558.80	0.119
**Post Crisis**	**Expected Percentage**	9.16	9.13	9.09	9.05	9.02	8.98	8.95	8.91	8.87	8.85		
	**Deviation Rate**	-61.51	5.10	8.64	11.68	10.87	5.63	5.10	6.71	5.18	3.61		
** **	**Z-Stat**	**17.77** **	0.84	1.39	**1.86** *	**1.73** *	0.91	0.83	1.08	0.84	0.59		

Note: Observed and expected values have been determined in the percentage of the sample (*N*). Deviation rate has been calculated by taking the difference of observed and expected percentage values divided by the expected percentage value.

*, ** and *** indicate significance at 10, 5 and 1% levels, respectively.

Table 10 presents the results for the pre- and post-crisis period in the context of the US based firms. The *z* statistic is significant at the numbers 1, 2 and 5 in the 1^st^ digit during the pre-crisis period. However, in the 1^st^ digit, no significant change is observed during the post-crisis period. In the 2^nd^ digit, the pre-crisis period is also showing insignificant change. In the 2^nd^ digit for the post-crisis period the *z* statistic is significant for the number 7. The positive deviation reveals that observed values are more frequent than expected values and negative deviation rate shows that observed frequencies are fewer than expected frequencies. In the 3^rd^ digit, during both the pre- and post-crisis period, the *z* statistic is significant for the numbers *zero* and 9. The value of the χ^2^-test illustrates that the 3^rd^ digit is statistically significant for the post-crisis period. Concluding, the results suggest that opportunities for manipulation of earnings have reduced with the strengthening of governance mechanism and laws especially after Sarbanes Oxley Act.

Table 11 reports the results for pre- and post-crisis period in the context of Pakistani listed firms. The *z* statistic is significant at numbers 6 and 7 in the 1^st^ digit during the pre-crisis period. However, in the 1^st^ digit, number 5 suggests a significant change in the post-crisis period. In the 2^nd^ digit, the pre-crisis period represents a significant change in the numbers *zero* and 1. In the 2^nd^ digit for post-crisis period, *z* statistic is significant for the number 2. The positive deviation reveals that observed values are more frequented than expected values, and vice versa. In the 3^rd^ digit, all the numbers except 1 have shown a significant change for earnings management during the pre- and post-crisis period. The value of the χ^2^-test at the 3^rd^ digit is statistically significant for the pre-crisis period. This suggests that, in Pakistan, managers have more incentives to manipulate the earnings at the 3^rd^ digit for the pre-crisis period, but after the crisis period the CEM behaviour is restrained. The results for the Cramer’s V are showing that all the digits (1 to 3) have more values for the pre-crisis period, so more variation is seen during the pre-crisis period in the context of Pakistan.

**Table 11:** Pre and post crisis (Pakistani listed firms) from 1^st^ to 3^rd^ digits Benford’s Law on net annual income of companies in Pakistan.

	Number	0	1	2	3	4	5	6	7	8	9	χ^2^	Cramer’s V
**1**^**st**^ **digit; *N* = 2405**	**Observed %**		30.6	18.05	12.56	10.48	8.69	5.7	4.91	4.86	4.16	13.05	0.026
**Pre-crisis**	**Expected %**		30.1	17.61	12.49	9.69	7.92	6.7	5.8	5.12	4.58		
	**Deviation rate**		1.67	2.47	0.54	8.13	9.73	-14.98	-15.41	-4.98	-9.21		
	***Z* statistic**		0.53	0.56	0.1	1.26	1.34	**2.12** **	**2.03** ^**^	0.58	1.04		
**1**^**st**^ **digit; *N* = 1460**	**Observed %**		31.1	16.92	13.49	9.59	6.3	6.92	6.37	4.25	5.07	12.2	0.032
**Post-crisis**	**Expected %**		30.1	17.61	12.49	9.69	7.92	6.7	5.8	5.12	4.58		
	**Deviation rate**		3.31	-3.93	8.03	-1.04	-20.44	3.25	9.83	-17.06	10.67		
** **	***Z* statistic**		0.82	0.71	1.12	0.13	**2.54** ^***^	0.33	0.89	**1.65** ^*^	0.85		
**2**^**nd**^ **digit; *N* = 2405**	**Observed %**	13.93	10.27	10.89	10.81	9.48	9.31	8.65	9.31	8.69	8.65	12.58	0.024
**Pre-crisis**	**Expected %**	11.97	11.39	10.88	10.43	10.03	9.67	9.34	9.04	8.76	8.5		
	**Deviation rate**	16.37	-9.83	0.13	3.65	-5.48	-3.68	-7.4	3.03	-0.8	1.75		
	**Z-Stat**	**2.77** ^***^	**1.81** ^*^	0.02	0.6	0.92	0.6	1.21	0.46	0.12	0.26		
**2**^**nd**^ **digit; *N* = 1460**	**Observed %**	11.78	11.58	9.45	10.68	10.07	10.27	8.84	8.77	9.45	9.11	6.11	0.022
**Post-crisis**	**Expected %**	11.97	11.39	10.88	10.43	10.03	9.67	9.34	9.04	8.76	8.5		
	**Deviation rate**	-1.58	1.63	-13.12	2.44	0.38	6.25	-5.4	-3.02	7.9	7.17		
** **	***Z* statistic**	0.22	0.22	**1.86** ^*^	0.31	0.05	0.76	0.68	0.37	0.9	0.81		
**3**^**rd**^ **digit; *N* = 2405**	**Observed %**	23.74	9.98	8.61	8.73	8.19	8.23	8.69	7.4	7.86	8.57	257.56***	0.109
**Pre-crisis**	**Expected %**	10.18	10.14	10.1	10.06	10.02	9.98	9.94	9.9	9.86	9.83		
	**Deviation rate**	133.22	-1.59	-14.78	-13.2	-18.25	-17.51	-12.57	-25.24	-20.3	-12.86		
	***Z* statistic**	**15.63** ^***^	0.33	**3.25** ^***^	**2.87** ^**^	**4.07** ^***^	**3.88** ^***^	**2.71** ^**^	**5.83** ^***^	**4.54** ^***^	**2.76** ^**^		
**3**^**rd**^ **digit; *N* = 1460**	**Observed %**	10.96	9.79	11.44	9.79	10.34	9.79	9.32	9.04	10	9.52	5.64	0.021
**Post-crisis**	**Expected %**	10.18	10.14	10.1	10.06	10.02	9.98	9.94	9.9	9.86	9.83		
	**Deviation rate**	7.65	-3.41	13.25	-2.64	3.22	-1.86	-6.29	-8.68	1.42	-3.15		
** **	***Z* statistic**	0.95	0.44	1.61	0.34	0.4	0.24	0.82	1.14	0.18	0.4		

Note: Observed and expected values have been determined in the percentage of the sample (*N*). Deviation rate has been calculated by taking the difference of observed and expected percentage values divided by the expected percentage value.

*, ** and *** indicate significance at 10, 5 and 1% levels, respectively.

Table 12 reported results are showing for pre and post crisis time period obtained by using positive net income of Indian based companies. Z-statistic is significant if 1^st^ digit is 1 and 4 to 7 during pre-crisis time period. However, in 1^st^ digit number only 3 is showing insignificant change in post crisis time period. In 2^nd^ digit pre-crisis time period, no one is showing significant results, but post crisis time period the results are significant if 2^nd^ digit is 0, 1 or 9. During pre-crisis time period, the results are significant if 3^rd^ digit is 0, 2, 5 or 9, however, after crisis time period the results are significant if 3^rd^ digit is 0 or 3.

**Table 12:** Pre and post crisis (Indian based companies) from 1st to 3rd digits Benford’s Law on net annual income companies in India.

	Number	0	1	2	3	4	5	6	7	8	9	Chi-Square	Cramer’s V
**1st digit n = 13119**	**Observed Percentage**		43.88	19.40	10.86	7.93	5.42	4.14	3.50	2.68	2.20	1860.44	0.133
**Pre-Crisis**	**Expected Percentage**		30.1	17.61	12.49	9.69	7.92	6.7	5.8	5.12	4.58		
	**Deviation Rate**		45.77	10.16	-13.03	-18.19	-31.57	-38.22	-39.68	-47.60	-52.07		
	**Z-Stat**		**9.69** ^***^	1.58	**1.83** ^*^	**2.28** ^**^	**3.86** ^**^	**4.49** ^***^	**4.37** ^***^	**5.26** ^***^	**5.68** ^***^		
**1st digit n = 11125**	**Observed Percentage**		40.95	19.73	11.26	8.12	5.95	4.63	3.64	3.36	2.38	1043.00	0.108
**Post Crisis**	**Expected Percentage**		30.1	17.61	12.49	9.69	7.92	6.7	5.8	5.12	4.58		
	**Deviation Rate**		36.04	12.04	-9.88	-16.22	-24.83	-30.96	-37.30	-34.43	-48.12		
** **	**Z-Stat**		**11.06** ^***^	**2.67** ^**^	**1.96** ^**^	**2.88** ^**^	**4.16** ^***^	**4.95** ^***^	**5.79** ^***^	**4.90** ^***^	**7.25** ^***^		
**2nd digit n = 13119**	**Observed Percentage**	10.91	10.00	9.99	9.94	10.02	10.15	10.15	9.72	9.63	9.49	80.38	0.026
**Pre-Crisis**	**Expected Percentage**	11.97	11.39	10.88	10.43	10.03	9.67	9.34	9.04	8.76	8.5		
	**Deviation Rate**	-8.88	-12.20	-8.16	-4.71	-0.11	4.98	8.69	7.48	9.90	11.70		
	**Z-Stat**	1.19	**1.62** ^*^	1.03	0.57	0.01	0.56	0.94	0.80	1.03	1.18		
**2nd digit n = 11125**	**Observed Percentage**	10.20	9.70	10.15	10.53	10.03	9.82	10.06	9.86	9.78	9.88	98.60	0.031
**Post Crisis**	**Expected Percentage**	11.97	11.39	10.88	10.43	10.03	9.67	9.34	9.04	8.76	8.5		
	**Deviation Rate**	-14.83	-14.86	-6.74	0.93	-0.01	1.59	7.69	9.11	11.59	16.27		
** **	**Z-Stat**	**2.94** ^**^	**2.87** ^**^	1.22	0.16	0.00	0.26	1.20	1.38	**1.71** ^*^	**2.32** ^**^		
**3rd digit n = 13119**	**Observed Percentage**	2.94	11.61	11.97	11.76	11.77	11.84	11.77	11.61	11.33	3.40	2596.00	0.148
**Pre-Crisis**	**Expected Percentage**	10.18	10.14	10.1	10.06	10.02	9.98	9.94	9.9	9.86	9.83		
	**Deviation Rate**	-71.09	14.49	18.52	16.87	17.50	18.63	18.36	17.26	14.90	-65.37		
	**Z-Stat**	**14.95** ^***^	**1.60** ^*^	**2.01** ^**^	**1.84** ^*^	**1.90** ^*^	**2.01** ^**^	**1.98** ^**^	**1.86** ^*^	**1.62** ^*^	**12.37** ^***^		
**3rd digit n = 11125**	**Observed Percentage**	3.22	10.71	11.07	11.37	11.23	10.60	10.50	10.64	10.43	10.22	1732.00	0.132
**Post Crisis**	**Expected Percentage**	10.18	10.14	10.1	10.06	10.02	9.98	9.94	9.9	9.86	9.83		
	**Deviation Rate**	-68.35	5.67	9.60	12.98	12.08	6.26	5.67	7.46	5.75	4.01		
** **	**Z-Stat**	**19.75** ^***^	0.93	1.55	**2.06** ^**^	**1.92** ^**^	1.02	0.92	1.20	0.93	0.65		

Note: Observed and expected values have been determined in the percentage of the sample (*N*). Deviation rate has been calculated by taking the difference of observed and expected percentage values divided by the expected percentage value

*, ** and *** indicate significance at 10, 5 and 1% levels, respectively.

Table 13 reported results are showing for pre and post crisis time period obtained by using positive net income of Chinese based companies. Z-statistic is significant if 1^st^ digit is 1 to 3, 8 and 9 in pre-crisis time period. However, in 1^st^ digit number only 4 is showing insignificant change in post crisis time period. In 2^nd^ digit the significant results are observed at 1, 2, 8 and 9 in both pre and post crisis period. Likewise, the results are significant if 3^rd^ digit is 1, 4, 6 to 9in both time periods. It means that Chinese firms are more inclined to rounding off at all the three digits. However, the phenomenon is slightly different during pre and post crisis period at 1^st^ digit level.

**Table 13:** Pre and post crisis (Chinese based companies) from 1st to 3rd digits Benford’s Law on net annual income of companies in China.

	Number	0	1	2	3	4	5	6	7	8	9	Chi-Square	Cramer’s V
**1st digit n = 11536**	**Observed Percentage**		42.58	5.10	15.19	11.48	8.45	6.36	4.28	3.79	2.76	4887.00	0.230
**Pre-Crisis**	**Expected Percentage**		30.1	17.61	12.49	9.69	7.92	6.7	5.8	5.12	4.58		
	**Deviation Rate**		41.47	-71.01	21.61	18.48	6.71	-5.06	-26.25	-25.98	-39.83		
	**Z-Stat**		**15.42** ^***^	**34.70** ^***^	**4.59** ^***^	**3.43** ^***^	1.17	0.85	**4.60** ^***^	**4.25** ^***^	**6.81** ^***^		
**1st digit n = 8650**	**Observed Percentage**		32.19	23.80	13.84	9.33	6.87	5.17	3.48	3.08	2.24	899.67	0.114
**Post Crisis**	**Expected Percentage**		30.1	17.61	12.49	9.69	7.92	6.7	5.8	5.12	4.58		
	**Deviation Rate**		6.93	35.16	10.79	-3.68	-13.25	-22.82	-40.04	-39.82	-51.09		
** **	**Z-Stat**		**2.73** ^**^	**8.88** ^***^	**2.38** ^**^	0.75	**2.53** ^**^	**4.22** ^***^	**7.74** ^***^	**7.21** ^***^	**9.66** ^***^		
**2nd digit n = 11536**	**Observed Percentage**	8.67	8.11	8.26	7.94	8.20	8.42	7.85	8.15	7.81	7.95	546.40	0.073
**Pre-Crisis**	**Expected Percentage**	11.97	11.39	10.88	10.43	10.03	9.67	9.34	9.04	8.76	8.5		
	**Deviation Rate**	-27.57	-28.83	-24.10	-23.91	-18.22	-12.98	-15.94	-9.86	-10.86	-6.49		
	**Z-Stat**	**7.16** ^***^	**7.35** ^***^	**5.82** ^***^	**5.64** ^***^	**4.07** ^***^	**2.76** ^***^	**3.38** ^***^	**1.99** ^**^	**2.16** ^**^	1.25		
**2nd digit n = 8650**	**Observed Percentage**	10.43	9.67	10.40	9.67	10.00	10.19	9.97	9.92	9.86	9.88	147.43	0.043
**Post Crisis**	**Expected Percentage**	11.97	11.39	10.88	10.43	10.03	9.67	9.34	9.04	8.76	8.5		
	**Deviation Rate**	-12.88	-15.10	-4.43	-7.28	-0.32	5.39	6.78	9.79	12.60	16.26		
** **	**Z-Stat**	**3.08** ^***^	**3.55** ^***^	0.96	1.57	0.07	1.05	1.29	**1.81** ^*^	**2.26** ^**^	**2.83** ^***^		
**3rd digit n = 11536**	**Observed Percentage**	0.20	8.98	9.23	8.75	9.05	9.41	8.58	9.12	9.07	8.91	124.30	0.035
**Pre-Crisis**	**Expected Percentage**	10.18	10.14	10.1	10.06	10.02	9.98	9.94	9.9	9.86	9.83		
	**Deviation Rate**	-97.99	-11.42	-8.63	-13.05	-9.70	-5.67	-13.69	-7.88	-7.99	-9.36		
	**Z-Stat**	**134.96** ^***^	**2.47** ^***^	**1.84** ^*^	**2.84** ^***^	**2.07** ^**^	1.18	**2.97** ^***^	**1.65** ^*^	**1.68** ^*^	**1.97** ^**^		
**3rd digit n = 8650**	**Observed Percentage**	0.22	11.13	11.16	10.98	11.07	11.49	10.48	11.19	11.06	11.22	7657.00	0.314
**Post Crisis**	**Expected Percentage**	10.18	10.14	10.1	10.06	10.02	9.98	9.94	9.9	9.86	9.83		
	**Deviation Rate**	-97.88	9.79	10.52	9.11	10.44	15.10	5.47	13.06	12.17	14.17		
** **	**Z-Stat**	131.01***	**1.93** ^*^	**2.06** ^**^	**1.79** ^*^	**2.04** ^**^	**2.89** ^***^	**1.08**	**2.50** ^***^	**2.34** ^**^	**2.69** ^***^		

Note: Observed and expected values have been determined in the percentage of the sample (*N*). Deviation rate has been calculated by taking the difference of observed and expected percentage values divided by the expected percentage value.

*, ** and *** indicate significance at 10, 5 and 1% levels, respectively.

Table 14 reported results are showing for pre and post crisis time period obtained by using positive net income of Brazilian companies. Z-statistic is significant if 1^st^ digit is 1 to 9 during pre-crisis time period. However, in 1^st^ digit number only 2 to 4 is showing insignificant change in post crisis time period. In 2^nd^ digit pre-crisis time period is showing significant results, but post crisis time period the results are significant if 2^nd^ digit is 0 to 4 and then 7 or 9. During pre-crisis time period, the results are significant if 3^rd^ digit is 0 to 7, however, after crisis time period the results are significant if 3^rd^ digit is from 4 or 9.

**Table 14:** Pre and post crisis (Brazilian based companies) from 1st to 3rd digits Benford’s Law on net annual income of companies in Brazil.

	Number	0	1	2	3	4	5	6	7	8	9	Chi-Square	Cramer’s V
**1st digit n = 1386**	**Observed Percentage**		13.44	6.14	28.54	45.45	3.03	1.16	0.72	0.58	0.87	2561.00	0.481
**Pre-Crisis**	**Expected Percentage**		30.1	17.61	12.49	9.69	7.92	6.7	5.8	5.12	4.58		
	**Deviation Rate**		-55.35	-65.12	128.51	369.02	-61.68	-82.75	-87.54	-88.71	-81.07		
	**Z-Stat**		**29.84** ^***^	**29.18** ^***^	**21.71** ^***^	**43.87** ^***^	**17.40** ^***^	**31.68** ^***^	**36.62** ^***^	**36.60** ^***^	**24.46** ^***^		
**1st digit n = 1576**	**Observed Percentage**		38.5	0	0	0	4.45	14.24	14.24	14.21	14.24	812.00	0.254
**Post Crisis**	**Expected Percentage**		30.1	17.61	12.49	9.69	7.92	6.7	5.8	5.12	4.58		
	**Deviation Rate**		27.91	-100.00	-100.00	-100.00	-43.78	112.51	145.48	177.62	210.88		
** **	**Z-Stat**		**10.54** ^***^	0.00	0.00	0.00	**10.27** ^***^	**13.18** ^***^	**14.75** ^***^	**15.91** ^***^	**16.88** ^***^		
**2nd digit n = 1386**	**Observed Percentage**	9.25	9.39	9.54	11.63	10.91	9.10	10.12	10.19	9.83	9.54	26.98	0.046
**Pre-Crisis**	**Expected Percentage**	11.97	11.39	10.88	10.43	10.03	9.67	9.34	9.04	8.76	8.5		
	**Deviation Rate**	-22.74	-17.53	-12.34	11.53	8.78	-5.85	8.30	12.70	12.18	12.21		
	**Z-Stat**	**5.74** ^***^	**4.18** ^***^	**2.79** ^**^	**2.29** ^**^	**1.72** ^*^	1.20	1.57	**2.32** ^**^	**2.19** ^**^	**2.16** ^**^		
**2nd digit n = 1576**	**Observed Percentage**	10.36	10.10	9.40	10.29	10.31	9.52	10.05	10.43	9.79	9.64	31.99	0.047
**Post Crisis**	**Expected Percentage**	11.97	11.39	10.88	10.43	10.03	9.67	9.34	9.04	8.76	8.5		
	**Deviation Rate**	-13.47	-11.37	-13.56	-1.38	2.79	-1.51	7.58	15.36	11.71	13.45		
** **	**Z-Stat**	**3.23** ^***^	**2.62** ^***^	**3.09** ^***^	0.29	0.56	0.30	1.44	**2.77** ^***^	**2.11** ^**^	**2.36** ^**^		
**3rd digit n = 1386**	**Observed Percentage**	0.29	11.05	12.14	11.13	10.98	10.84	11.05	10.91	10.69	10.77	4697.86	0.614
**Pre-Crisis**	**Expected Percentage**	10.18	10.14	10.1	10.06	10.02	9.98	9.94	9.9	9.86	9.83		
	**Deviation Rate**	-97.16	9.02	20.19	10.61	9.61	8.60	11.22	10.21	8.45	9.52		
	**Z-Stat**	**112.54** ^***^	**1.78** ^*^	**3.81** ^***^	**2.07** ^**^	**1.88** ^*^	**1.69** ^*^	**2.17** ^*^	**1.98** ^*^	**1.65** ^*^	**1.84** ^*^		
**3rd digit n = 1576**	**Observed Percentage**	0.00	10.76	11.33	11.02	11.14	11.31	11.31	10.50	11.07	11.52	22.04	0.039
**Post Crisis**	**Expected Percentage**	10.18	10.14	10.1	10.06	10.02	9.98	9.94	9.9	9.86	9.83		
	**Deviation Rate**	-100.00	6.13	12.21	9.58	11.21	13.32	13.78	6.06	12.29	17.23		
** **	**Z-Stat**	0.00	1.23	**2.38** ^**^	**1.88** ^*^	**2.18** ^**^	**2.56** ^***^	**2.64** ^***^	1.20	**2.36** ^**^	**3.24** ^***^		

Note: Observed and expected values have been determined in the percentage of the sample (*N*). Deviation rate has been calculated by taking the difference of observed and expected percentage values divided by the expected percentage value.

*, ** and *** indicate significance at 10, 5 and 1% levels, respectively.

Table 15 reported results are showing for pre and post crisis time period obtained by using positive net income of Indian based companies. Z-statistic is significant if 1^st^ digit is 1 to 4, 6 and 9 during pre-crisis time period. However, in 1^st^ digit number only 0, 4 and 6 are showing significant change in post crisis time period. In 2^nd^ digit pre-crisis time period, no one is showing significant results, but post crisis time period the results are insignificant if 2^nd^ digit is 5 or 7. During pre-crisis time period, the results are insignificant if 3^rd^ digit is 1, 5 or 6, however, after crisis time period the results are significant if 3^rd^ digit is 1, 4 or 9.

**Table 15:** Pre and post crisis (Russian based companies) from 1st to 3rd digits Benford’s Law on net annual income of companies in Russia.

	Number	0	1	2	3	4	5	6	7	8	9	Chi-Square	Cramer’s V
**1st digit n = 216**	**Observed Percentage**		31.10	20.0	18.10	7.90	6.50	6.94	4.20	3.24	1.85	9.63	0.075
**Pre-Crisis**	**Expected Percentage**		30.10	17.61	12.49	9.69	7.92	6.70	5.80	5.12	4.58		
	**Deviation Rate**		0.03	13.05	44.56	-18.78	-18.16	3.65	-28.16	-36.70	-59.57		
	**Z-Stat**		0.01	**3.51** ^***^	**8.84** ^***^	**4.13** ^***^	**3.57** ^***^	0.59	**4.99** ^***^	**6.48** ^***^	**12.36** ^***^		
**1st digit n = 1379**	**Observed Percentage**		32.27	18.35	12.33	7.54	7.83	5.73	6.24	5.37	4.28	13.74	0.035
**Post Crisis**	**Expected Percentage**		30.1	17.61	12.49	9.69	7.92	6.70	5.80	5.12	4.58		
	**Deviation Rate**		7.21	4.18	-1.30	-22.17	-1.11	-14.50	7.52	4.81	-6.58		
** **	**Z-Stat**		**2.83** ^***^	1.16	0.30	**4.97** ^***^	0.20	**2.55** ^***^	1.10	0.67	0.91		
**2nd digit n = 216**	**Observed Percentage**	9.26	10.65	6.94	9.26	12.04	12.04	7.87	9.72	9.26	9.72	9.44	0.070
**Pre-Crisis**	**Expected Percentage**	11.97	11.39	10.88	10.43	10.03	9.67	9.34	9.04	8.76	8.5		
	**Deviation Rate**	-22.65	-6.51	-36.17	-11.22	20.01	24.48	-15.73	7.55	5.70	14.38		
	**Z-Stat**	**5.71** ^***^	1.47	**9.46** ^***^	**2.47** ^**^	**3.77** ^***^	**4.44** ^***^	**3.33** ^***^	1.41	1.05	**2.52** ^***^		
**2nd digit n = 1379**	**Observed Percentage**	10.95	9.50	9.14	9.14	11.24	9.06	10.44	9.06	9.93	9.93	18.38	0.038
**Post Crisis**	**Expected Percentage**	11.97	11.39	10.88	10.43	10.03	9.67	9.34	9.04	8.76	8.5		
	**Deviation Rate**	-8.52	-16.60	-16.02	-12.40	12.06	-6.26	11.80	0.27	13.41	16.88		
** **	**Z-Stat**	**2.00** ^**^	**3.94** ^***^	**3.69** ^***^	**2.74** ^***^	**2.34** ^**^	1.29	**2.20** ^**^	0.05	**2.40** ^**^	**2.93** ^***^		
**3rd digit n = 216**	**Observed Percentage**	0.93	10.65	13.43	7.87	6.02	10.65	10.65	7.87	14.81	16.20	22.04	0.106
**Pre-Crisis**	**Expected Percentage**	10.18	10.14	10.1	10.06	10.02	9.98	9.94	9.9	9.86	9.83		
	**Deviation Rate**	-90.90	5.01	32.93	-21.77	-39.93	6.69	7.12	-20.50	50.25	64.84		
	**Z-Stat**	**59.02** ^***^	1.01	**5.96** ^***^	**4.97** ^***^	**10.28** ^***^	1.32	1.40	**4.60** ^***^	**8.52** ^***^	**10.57** ^***^		
**3rd digit n = 1379**	**Observed Percentage**	5.00	10.88	10.37	9.79	11.39	10.30	10.22	9.35	12.04	10.51	20.83	0.041
**Post Crisis**	**Expected Percentage**	10.18	10.14	10.1	10.06	10.02	9.98	9.94	9.9	9.86	9.83		
	**Deviation Rate**	-50.85	7.27	2.67	-2.69	13.62	3.18	2.87	-5.51	22.09	6.97		
** **	**Z-Stat**	**14.50** ^***^	1.45	0.54	0.56	**2.62** ^***^	0.64	0.57	1.14	**4.09** ^***^	1.36		

Note: Observed and expected values have been determined in the percentage of the sample (*N*). Deviation rate has been calculated by taking the difference of observed and expected percentage values divided by the expected percentage value.

*, ** and *** indicate significance at 10, 5 and 1% levels, respectively.

To sum up, it is observed that both the emerging economies have more variations in net income’s all digits (1^**st**^ to 3^**rd**^). However, the results are in consistent with the previous study Lin & Wu [44] that the managers from both emerging and developed countries are engaged in cosmetic earnings but in emerging economies hold more incentive for managers.

## 5. Conclusion

This study analyzes the earnings management practices of listed companies in UK, US, Brazilian, Russian, Indian, Chinese and Pakistani firms using Benford’s law. We observe that corporations of both developed and emerging markets engage in earnings management. Generally, it is believed that managers in developed markets have fewer opportunities to manipulate earnings due to the presence of strong governance and monitoring mechanism (Li, 2019). Similarly, the results from this study also suggest that earnings management is practiced more in emerging markets than in developed markets. Hence Hypothesis 1 is accepted.

The Enron, World Call, and Tyco financial scandals raised serious concerns over the credibility of the financial system. The existing literature suggests that corporate governance mechanisms restrict managers’ earnings manipulation behavior. Therefore, in order to strengthen governance mechanisms, various countries introduced and improved their regulatory frameworks. The results of current research show that strengthening governance mechanisms reduced earnings management in both developed and emerging markets after the global financial crisis. However, developed markets are more effective at controlling cosmetic earnings management behavior than are emerging markets. Therefore, Hypothesis 2 is accepted. Finally, the results suggest that managers of both the developed and developing countries are engaged in earnings management after the global financial crisis. However, the intensity of its use is higher in developing and emerging economies. The empirical findings are useful for regulatory authorities, investors, and other stakeholders as they shed light on the restriction of managerial behavior due to improvements to the governance mechanisms.

The need for better governance and legal systems to safeguard the interest of stakeholders increased manifold in the last decade. Moreover, financial statements are becoming more flexible with the international adoption of International Financial Reporting Standards (IFRS). Therefore, we urge regulators to improve governance and legislative frameworks to restrict earnings management especially in emerging markets. Further, while making an investment in emerging markets, investors should use their due diligence to analyze the true and fair representation of financial information.
